# Association between post-intravenous thrombolysis delirium and 90-day functional outcomes in patients with acute ischemic stroke

**DOI:** 10.3389/fpsyt.2026.1879174

**Published:** 2026-09-03

**Authors:** Yanan Hao, Fei Wu, Yunzhu Tang, Xuemei Shi, Xiaodan Yang, Xiaoling Zhang

**Affiliations:** 1 JiaXing Key Laboratory of Fundamental and Clinical Research on Cerebral Protection in Acute Ischemic Cerebrovascular Disease (A), Department of Neurology, The Second Affiliated Hospital of Jiaxing University, Jiaxing, Zhejiang, China; 2 Department of Neurology, Weinan Central Hospital Hospital, Weinan, Shaanxi, China; 3 Jiaxing University affiliated to Zhejiang Chinese Medicine University, Hangzhou, Zhejiang, China; 4 Department of Hepatobiliary Surgery, The Second Affiliated Hospital of Jiaxing University, Jiaxing, Zhejiang, China

**Keywords:** delirium, dysphagia, ischemic stroke, reperfusion, therapy, intravenous thrombolysis

## Abstract

**Objective:**

This study aimed to examine the association between post-Intravenous thrombolysis delirium and 90-day functional outcomes in patients with acute ischemic stroke (AIS).

**Methods:**

Patients admitted to the Second Hospital of Jiaxing within 24 hours of symptom onset who underwent intravenous thrombolysis for AIS between January 1, 2023, and December 31, 2024, were retrospectively analyzed. Clinical data were collected to evaluate factors associated with the occurrence of post-reperfusion delirium. Functional outcomes at 90 days were assessed using the modified Rankin Scale, with scores of 0–2 classified as favorable and scores of 3–6 classified as poor. Multivariate logistic regression analyses were performed to identify independent risk factors for the development of post-reperfusion delirium and unfavorable 90-day functional outcomes.

**Results:**

A total of 272 patients were included, comprising 173 males and 99 females, with a mean age of 67.27 ± 11.74 years. Delirium developed in 42 patients, corresponding to an incidence rate of 15.4%. Dysphagia (OR 22.695, 95% CI 9.853 to 52.135, P < 0.001), secondary infection (OR 18.039, 95% CI 8.713 to 37.351, P < 0.001), and hypoproteinemia (OR 9.168, 95% CI 4.514 to 18.618, P < 0.001) were strong independent risk factors for delirium in acute cerebral infarction. Age, illiteracy, increased baseline NIHSS score, the use of sedative drugs, and TOAST classification as large artery atherosclerosis type/cardiogenic embolism type would significantly increase the risk of delirium, and delirium was highly correlated with short-term mortality risk. Delirium was a strong independent risk factor for poor functional prognosis 90 days after cerebral infarction (OR = 12.356, 95% CI 5.892 to 26.008, P < 0.001). At the same time, increasing age, hypertension, multiple comorbidities after admission (electrolyte imbalance, anemia, hypoproteinemia, dysphagia, secondary infection, etc.), increased baseline NIHSS score, and TOAST classification as large artery atherosclerosis type/cardiogenic embolism type would also significantly increase the risk of poor functional prognosis 90 days after cerebral infarction in patients.

**Conclusion:**

Delirium is significantly associated with poor functional outcomes at 90 days in patients with AIS treated with intravenous thrombolysis, highlighting the clinical importance of early delirium detection. Dysphagia, secondary infections, hypoproteinemia, high baseline NIHSS scores, and large-artery atherosclerotic and cardioembolic stroke subtypes are independently associated with both delirium occurrence and adverse 90-day outcomes.

## 1 Introduction

With the aging of the Chinese population, stroke has emerged as a major public health concern with significant implications for physical health. Among all stroke types, ischemic cerebral infarction remains the most prevalent, accounting for approximately 69.6%–70.8% of all cases in China (1). Currently, early reperfusion therapy, including intravenous thrombolysis and endovascular therapy, represents the most effective treatment approach for ischemic stroke. Despite these advancements, a proportion of patients continue to experience unfavorable outcomes. A prospective study by Pasińska et al., which included 682 patients, indicated that those who developed delirium after stroke exhibited higher mortality rates compared to those who did not (2). These findings indicate a close association between post-stroke delirium (PSD) and prognosis in patients with AIS.

The incidence of delirium in stroke units varies between 10% and 50% (3), and it is mainly related to the patient’s age, type of stroke, and severity of the stroke. Reperfusion treatment may further increase the risk of delirium due to hemodynamic fluctuations. Studies have found that the incidence of delirium in patients undergoing reperfusion treatment ranges from 41% to 55% (4, 5), and patients with a NIHSS score of ≥ 10 and elderly patients are more likely to develop delirium (6). However, many domestic studies on reperfusion treatment have not reported the incidence of delirium. Moreover, studies led by China, such as extending the thrombolysis time window to 24 hours (TRACE-III) and using new thrombolytic drugs (reteplase), have not evaluated the risk of delirium. Several observational studies have found that delirium can increase the risk of disability by two times and the incidence of post-discharge cognitive impairment by 40% (7–9). Patients with delirium after reperfusion treatment have a significantly higher rate of deterioration in the 90-day modified Rankin Scale (mRS) score (OR = 3.2) (10). However, domestic studies on the prognosis of reperfusion treatment focus on physiological indicators (such as mRS, BI score), lacking analysis of the impact of delirium on long-term cognition (11, 12). This finding stands in marked contrast to the large population of ischemic stroke patients receiving reperfusion therapy in China.

Therefore, our study analyzed the clinical data of patients with AIS who received intravenous thrombolysis, aiming to explore the risk factors influencing the occurrence of delirium and prognosis after intravenous thrombolysis in AIS. The goal was to identify the risk factors for delirium after thrombolysis early, and to assess the association between delirium and the 90-day functional outcome. This study provides a basis for improving the clinical management and prognosis of patients with AIS who received intravenous thrombolysis.

## 2 Methods

### 2.1 Study participants

Patients with AIS who underwent intravenous thrombolysis and were admitted to the Second Hospital of Jiaxing within 24 hours of symptom onset between January 1, 2023, and December 31, 2024, were included in this study.

Inclusion criteria: (1) Age ≥ 18 years; (2) diagnosis of AIS; and (3) initiation of Intravenous thrombolysis within 24 hours of onset.

Exclusion criteria: (1) history of delirium or psychiatric disorders; (2) persistent coma; (3) severe aphasia; (4) imaging findings show that the lesion involves both the anterior and posterior circulation; (5) refusal to undergo scale assessments during hospitalization; and (6) loss to follow-up within 90 days.

### 2.2 Clinical data

Demographic characteristics, including age, sex, and educational level, were collected for all enrolled patients. Medical history variables included smoking, alcohol consumption, hypertension, diabetes, hyperlipidemia, coronary heart disease, and chronic obstructive pulmonary disease. Additional clinical variables included the number of prior stroke episodes, presence of dysphagia, baseline National Institutes of Health Stroke Scale (NIHSS) score upon admission, infarct location (anterior or posterior circulation), infarct etiology according to the Trial of Org 10172 in Acute Stroke Treatment (TOAST) classification, electrolyte and metabolic disturbances during hospitalization, anemia, hepatic insufficiency, renal insufficiency, hypoproteinemia, infection, use of sedative drugs, use of physical restraints, caregiver presence, hospital stay duration, and 90-day functional outcomes. This center has a relatively small number of patients undergoing mechanical thrombectomy, and the factors influencing the prognosis of mechanical thrombectomy are complex. Therefore, when including patients, those undergoing mechanical thrombectomy were excluded, so the reperfusion status was not included. For the patients undergoing intravenous thrombolysis, the pre-stroke mRS scores were all 0-1, so they were not included in the prognosis analysis. The information on thrombolysis bleeding transformation and infarction volume was incomplete, making it impossible to extract unified data. There were no unified standardized imaging measurement data for imaging-related markers. The occlusion sites were classified as large artery type and small artery type in the TOST classification, so these factors were not included in the analysis of the relationship between delirium and stroke prognosis.

Primary outcome measure: Functional outcomes at 90 days were evaluated using the modified Rankin Scale (mRS). A score of 0–2 was defined as a favorable outcome, while a score of 3–6 was defined as a poor outcome (13).

### 2.3 Assessment of delirium scales and clinical indicators

Delirium assessment was conducted using the Confusion Assessment Method (CAM) and the 4A Test (4AT) (14).

The CAM assesses eleven domains: onset pattern, attention deficits, thought disturbances, level of consciousness, disorientation, memory impairment, perceptual disturbances, psychomotor agitation or retardation, fluctuations, and alterations in the sleep–wake cycle. The total score ranges from 11 to 44 points. Scores below 20 indicate the absence of delirium, scores between 20 and 22 suggest possible delirium, and scores above 22 confirm delirium (15).

The 4AT evaluates four domains: alertness, orientation, attention, and the presence of acute changes with a fluctuating course. The total score ranges from 0 to 12 points. A score of ≥ 4 indicates possible delirium, scores between 1 and 3 indicate cognitive dysfunction, and a score of 0 indicates no delirium or severe cognitive dysfunction (16).

The diagnostic criteria for delirium are met if one of the diagnostic criteria in the two scales is satisfied: CAM score > 22 or 4AT score ≥ 4.

All patients received baseline delirium assessment on admission, followed by a repeated evaluation within 72 hours after thrombolysis. Dynamic surveillance was performed throughout this period. Immediate assessment would be conducted once patients presented with altered consciousness, impaired attention, disorganized thinking or fluctuating mental status. The monitoring period lasted from admission to 72 hours post thrombolysis. The subtype of delirium and corresponding clinical managements were documented.

#### 2.3.1 Electrolyte and metabolic disturbance

These disturbances were identified based on serum potassium (3.5–5.5 mmol/L) and sodium (135–145 mmol/L) concentrations. Values outside these ranges were considered abnormal.

#### 2.3.2 Anemia

Anemia was defined as hemoglobin levels below 120 g/L in males and below 110 g/L in females.

#### 2.3.3 Hepatic insufficiency

Hepatic insufficiency was determined that during hospitalization, alanine aminotransferase and aspartate aminotransferase were more than three times the baseline values.

#### 2.3.4 Renal insufficiency

Renal insufficiency was defined that during the hospitalization, the creatinine level was more than twice the baseline value.

#### 2.3.5 Hypoproteinemia

Hypoproteinemia was identified when serum total protein was below 60 g/L or serum albumin was below 35 g/L.

#### 2.3.6 Moderate to severe infection

Present with systemic symptoms (high fever, body temperature ≥ 38.5 °C, accompanied or not by chills, fatigue, listlessness, shortness of breath or consciousness disorders, etc.), elevated inflammatory indicators (white blood cell count ≥ 15x109/L and/or CRP ≥ 50mg/L and/or procalcitonin ≥ 0.5ng/ml), and treated with antibiotics.

DNT: Time from admission to thrombolysis.

Numerous studies have shown that dysphagia may indirectly increase the risk of delirium through pathways such as increased aspiration pneumonia, malnutrition, and psychological stress.

Hypoproteinemia, the presence or absence of a caregiver, and physical restraint are listed as predisposing factors for delirium in the Chinese Expert Consensus on Delirium in Clinical Settings.

The TOAST classification is mainly used for etiological classification of cerebral infarction. Different subtypes of cerebral infarction have varying overall severity of the condition and risk of complications, which may affect the occurrence of delirium. Although there are no large-scale literatures that directly compare the TOAST classification with the incidence of delirium, clinical practice tells us that the more severe the condition and the more complications, the higher the risk of delirium.

Therefore, dysphagia, hypoproteinemia, and TOAST classification are included as variables affecting delirium after cerebral infarction for analysis.

### 2.4 Data collection and management

Two researchers independently screened patients based on the predefined inclusion and exclusion criteria. In case of inconsistent results, a third senior researcher made the final decision.

All data were independently double-entered by two researchers using Microsoft Excel and cross-checked for consistency. Any inconsistencies were traced back to the original medical records for verification and correction. Abnormal values (such as data exceeding the reasonable physiological range) and missing values were checked. For cases with missing key variables, we attempted to supplement through telephone follow-ups. All data were anonymized and stored in a password-protected computer, accessible only to the research team members, to protect patient privacy.

### 2.5 Delirium assessment protocol and inter-rater reliability

Delirium was independently assessed by two trained neurologists with rich experience in stroke bedside evaluation. All assessors received unified standardized training on the Confusion Assessment Method (CAM) and the 4A Test (4AT), including theoretical learning and case simulation, to ensure consistent judgment criteria.

Strict blinding was applied throughout the study. The assessors were blinded to patients’baseline data, thrombolysis information and functional outcomes. In addition, the researchers responsible for outcome follow-up were unaware of the delirium evaluation results.

Inter-rater reliability was verified before formal enrollment. Thirty patients were randomly selected for double-blind independent evaluation, with a Kappa value of 0.87, indicating excellent inter-rater consistency. Regular double assessments were performed during the study to maintain stable evaluation quality.

### 2.6 Ethics statement

This study was approved by the Ethics Committee of the Second Affiliated Hospital of Jiaxing University. All procedures were conducted in accordance with the principles of the Declaration of Helsinki. Informed consent was obtained from all patients or their legal representatives for acceptance or refusal of intravenous thrombolysis or mechanical thrombectomy.

### 2.7 Statistical methods

Data analyses were performed using SPSS version 22.0 (IBM Corp., Armonk, NY, USA). The incidence of delirium and related risk factors was calculated. Continuous variables with a normal distribution were expressed as mean ± standard deviation (*SD*) and compared using independent-samples t-tests. Non-normally distributed continuous variables were presented as quartiles and compared using the rank-sum test. Categorical variables were summarized as frequencies and percentages and compared using Pearson’s chi-square test. The variables with p < 0.10 in the single-factor analysis, as well as the clinically significant variables (such as age, sex, NIHSS) were included in the multivariate Logistic regression analysis to screen out the independent risk factors for delirium and poor prognosis after intravenous thrombolysis in AIS. P value less than 0.05 indicates a statistically significant difference.

## 3 Results

**Figure 1:**
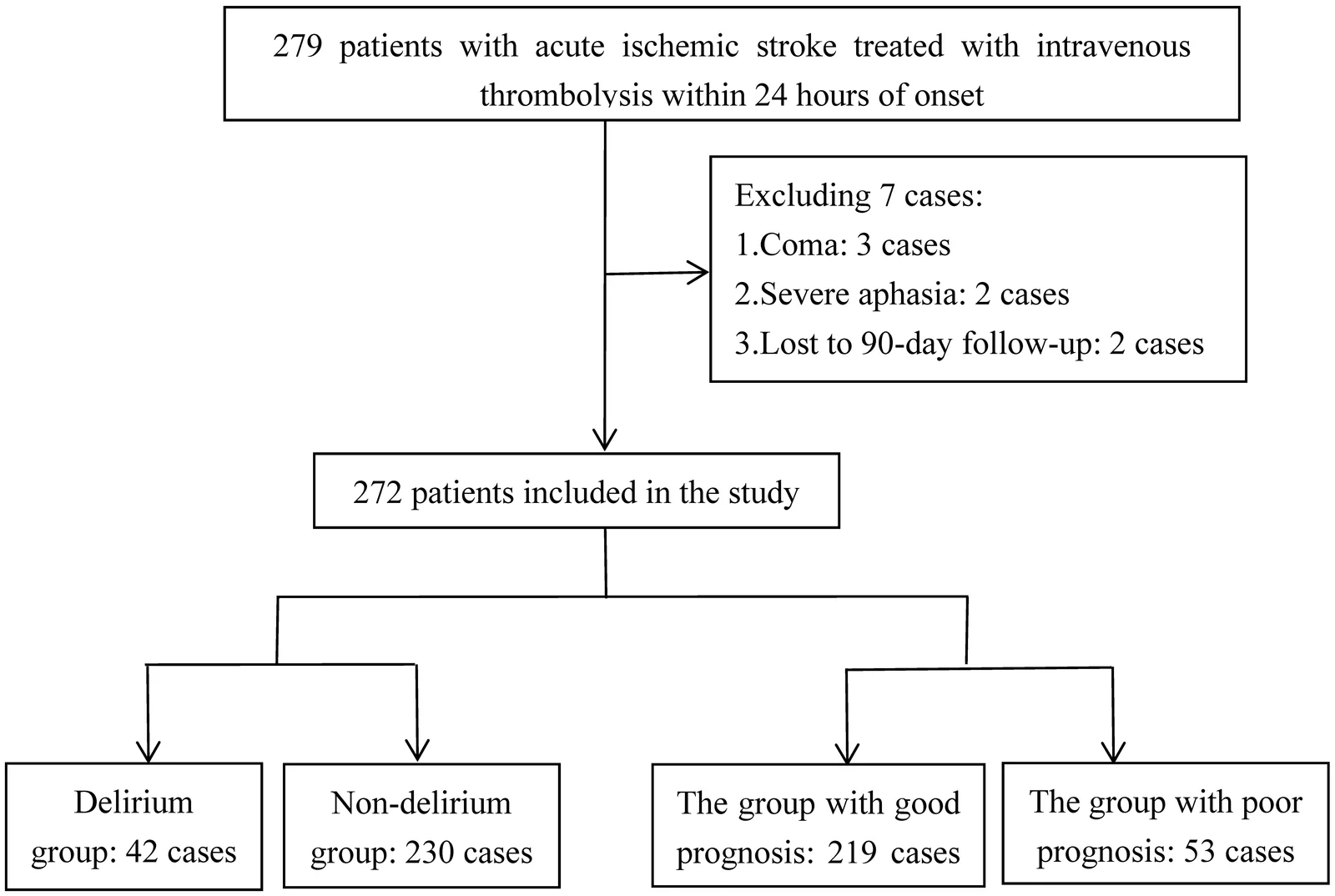
Study flowchart illustrating the inclusion and exclusion process for patients with AIS undergoing Intravenous thrombolysis.

During the study period, 279 patients with AIS who received Intravenous thrombolysis within 24 hours of onset were initially screened. After excluding 7 patients due to coma (*n* = 3), severe aphasia (*n* = 2) and loss to follow-up at 90 days (*n* = 2), a total of 272 patients were included in the final analysis. Among these, 42 patients developed delirium, and 230 did not. Based on 90-day mRS scores, 219 patients achieved favorable functional outcomes, while 53 had poor outcomes.The mean age of enrolled patients was 67.27 ± 11.74 years, comprising 173 males and 99 females. Study flowchart illustrating the inclusion and exclusion process for patients with AIS undergoing intravenous thrombolysis (**Figure 1**).

### 3.1 Univariate analysis of delirium development following intravenous thrombolysis in AIS

As presented in **Table 1**, Compared with the non-delirium group, the delirium group had a higher age (P < 0.001), a higher proportion of illiterates (P = 0.016), a higher proportion of patients with electrolyte disorders, anemia, liver dysfunction, hypoproteinemia, infection, and dysphagia after admission (all P < 0.05), a lower rate of accompanying caregivers after admission (P = 0.001), a higher baseline NIHSS score (all P < 0.001), and in the TOAST etiological classification, the delirium group had a higher proportion of large artery atherosclerosis type and cardiac embolism type, and a lower proportion of small artery occlusion type (P < 0.001). The proportion of patients using sedative drugs in the delirium group was higher (P < 0.001), and the mortality rate during hospitalization and 90 days after discharge was higher (all P < 0.05).

**Table 1:** Univariate analysis of factors associated with delirium following intravenous thrombolysis in patients with AIS.

Variable	Delirium group (*n* = 42)	Non-delirium group (*n* = 230)	*p*-value
Age (years)	75.19±9.31	65.83±11.40	<0.001
Sex (Male)	28 (63.6%)	145 (63.0%)	0.576
Illiterate (case)	23 (54.7%)	81 (35.2%)	0.016
Medical history	–	–	–
Smoking (case)	14 (33.3%)	77 (33.4%)	0.703
Alcohol abuse (case)	12 (28.5%)	56 (24.3%)	0.405
Hypertension (case)	30 (71.4%)	139 (60.4%)	0.456
Diabetes mellitus (case)	11 (26.1%)	63 (27.3%)	0.842
Hyperlipidemia (case)	8 (19.0%)	71 (30.8%)	0.429
Atrial fibrillation (case)	9 (21.4%)	22 (9.5%)	0.231
TIA / Cerebral infarction (case)	6 (14.2%)	39 (16.9%)	0.622
Chronic obstructive pulmonary disease (case)	1 (2.4%)	4 (1.7%)	0.565
Comorbidities after admission	–	–	–
Electrolyte and metabolic disturbance (case)	26 (61.9%)	52 (22.6%)	<0.001
Anemia (case)	21 (50.0%)	27 (11.7%)	<0.001
Hepatic insufficiency (case)	9 (21.4%)	8 (3.2%)	0.005
Renal insufficiency (case)	7 (16.6%)	11 (4.7%)	0.120
Hypoproteinemia (case)	31 (73.8%)	66 (28.6%)	<0.001
Infection (case)	30 (71.4%)	24 (10.4%)	<0.001
Dysphagia (case)	23 (54.7%)	8 (3.4%)	<0.001
Caregiver (case)	40 (95.2%)	229 (99.5%)	0.047
Physical restraint (case)	3 (7.1%)	12 (5.2%)	0.616
Reperfusion factors	–	–	–
Anterior circulation infarction (case)	34 (80.9%)	171 (74.3%)	0.361
Baseline NIHSS score	7 (3-12)	3 (2-4)	<0.001
DNT (min)	31.5 (25-44.5)	30 (24-36)	0.425
TOAST classification	**-**	**-**	<0.001
Large artery atherosclerosis (case)	22 (52.3%)	65 (28.2%)	–
Small artery occlusion (case)	7 (16.6%)	126 (54.7%)	–
Cardioembolism (case)	12 (28.5%)	24 (10.4%)	–
Stroke of other determined etiology (case)	0	4 (1.7%)	–
Stroke of undetermined etiology (case)	1 (2.3%)	11 (4.7%)	–
The use of sedative drugs (case)	18 (42.8%)	14 (6.0%)	<0.001
Death during hospitalization (case)	2 (4.8%)	0	0.037
Death within 90 days (case)	4 (9.5%)	0	0.027

TIA, Transient Ischemic Attack; DNT, door to needle time; ONT, onset to needle time; DPT, door to puncture time; TOAST, Trial of Org 10172 in Acute Stroke Treatment; mRS, modified Rankin Scale score; NIHSS, National Institutes of Health Stroke Scale.

### 3.2 Multivariable logistic regression analysis

As presented in **Table 2**, Logistic multivariate analysis revealed that dysphagia (OR 22.695, 95% CI 9.853 to 52.135, P < 0.001), secondary infection (OR 18.039, 95% CI 8.713 to 37.351, P < 0.001), and hypoproteinemia (OR 9.168, 95% CI 4.514 to 18.618, P < 0.001) were strong independent risk factors for delirium in acute cerebral infarction; age (OR 1.083, 95% CI 1.039 to 1.128, P < 0.001), illiteracy (OR 2.499, 95% CI 1.187 to 5.259, P = 0.016), increased baseline NIHSS score (OR 1.167, 95% CI 1.100 to 1.239, P < 0.001), the use of sedative drugs (OR 7.586, 95% CI 3.456 to 16.700, P < 0.001), and TOAST classification as large artery atherosclerosis type (OR 2.685, 95% CI 1.362 to 5.292, P = 0.004)/cardiogenic embolism type (OR 3.338, 95% CI 1.516 to 7.356, P = 0.003) all significantly increased the risk of delirium, and delirium was highly correlated with short-term mortality risk.

**Table 2:** Multivariate logistic regression analysis of independent risk factors for post-intravenous thrombolysis delirium in patients with AIS.

Variable	OR	95% CI	*p*-value
Age	1.083	1.039-1.128	<0.001
Sex	0.978	0.454-2.109	0.956
Illiterate	2.499	1.187-5.259	0.016
Electrolyte and Metabolic disturbance	1.325	0.419-4.194	0.632
Anemia	1.165	0.322-4.209	0.816
Hepatic insufficiency	1.764	0.289-10.451	0.532
Renal insufficiency	1.140	0.212-6.144	0.878
Hypoproteinemia	9.168	4.514-18.618	<0.001
Infection	18.039	8.713-37.351	<0.001
Dysphagia	22.695	9.853-52.135	<0.001
Caregiver	0.099	0.001-5.734	0.357
The use of sedative drugs	7.586	3.456-16.700	<0.001
DNT	1.005	0.996-1.014	0.287
Baseline NIHSS score	1.167	1.100-1.239	<0.001
TOAST classification (0 = small-artery occlusion)	**-**	**-**	**-**
Large-artery atherosclerotic	2.685	1.362-5.292	0.004
Cardioembolism	3.338	1.516-7.356	0.003
Stroke of other determined etiology	–	–	–
Stroke of undetermined etiology	–	–	–
The use of sedative drugs	49.018	2.105-1140.852	0.004
Death during hospitalization	60.789	2.765~1338.125	0.010

Variables with p < 0.1 in the univariate analysis were included in the logistic regression analysis.

TIA, Transient Ischemic Attack; TOAST, Trial of Org 10172 in Acute Stroke Treatment; mRS, modified Rankin Scale score; NIHSS, National Institutes of Health Stroke Scale.

### 3.3 Univariate analysis of factors influencing 90d functional outcomes in patients undergoing Intravenous thrombolysis for AIS

As presented in **Table 3**, Compared with the group with good prognosis, the group with poor prognosis had a higher age (P = 0.001), a higher proportion of previous hypertension (P = 0.026), and a higher incidence of electrolyte imbalance, anemia, liver dysfunction, hypoproteinemia, infection, dysphagia, and delirium after admission (all P < 0.05), a higher baseline NIHSS score, and a longer DNT time (all P < 0.05). In terms of TOAST etiological classification, the proportion of large artery atherosclerosis type and cardiogenic embolism type was higher in the group with poor prognosis, while the proportion of small artery occlusion type was lower (P < 0.001).

**Table 3:** Univariate analysis of factors influencing poor 90-day functional outcomes in patients with AIS after Intravenous thrombolysis.

Variable	90d mRS 0-2 (*n* = 219)	90d mRS 3-6 (*n* = 53)	*p*-value
Age (years)	66.09±11.14	72.17±12.85	0.001
Sex (Male)	134 (61.1%)	39 (73.5%)	0.092
Illiterate (case)	76 (34.7%)	25 (47.1%)	0.092
Medical history	–	–	–
Smoking (case)	75 (34.2%)	16 (30.1%)	0.406
Alcohol abuse (case)	50 (22.8%)	18 (33.9%)	0.093
Hypertension (case)	129 (58.9%)	40 (75.4%)	0.026
Diabetes mellitus (case)	61 (27.8%)	13 (24.5%)	0.625
Hyperlipidemia (case)	65 (29.6%)	14 (26.4%)	0.638
Coronary heart disease/atrial fibrillation (case)	21 (9.5%)	10 (18.8%)	0.056
TIA / Cerebral infarction (case)	38 (17.3%)	7 (13.2%)	0.466
Chronic obstructive pulmonary disease (case)	3 (1.3%)	1 (1.8%)	1.000
Comorbidities during hospitalization	–	–	–
Electrolyte and metabolic disturbance (case)	51 (23.2%)	27 (50.9%)	<0.001
Anemia (case)	27 (12.3%)	21 (39.6%)	<0.001
Hepatic insufficiency (case)	8 (3.6%)	9 (16.9%)	0.001
Renal insufficiency (case)	12 (5.4%)	6 (11.3%)	<0.001
Hypoproteinemia (case)	66 (30.1%)	31 (58.4%)	<0.001
Infection (case)	28 (12.7%)	26 (49.0%)	0.001
Dysphagia (case)	7 (3.1%)	24 (45.2%)	<0.001
Caregiver (case)	218 (99.5%)	51 (96.2%)	0.959
Physical restraint (case)	12 (5.4%)	3 (5.6%)	0.959
Delirium (case)	11 (5.0%)	31 (58.5%)	0.001
The use of sedative drugs (case)	12 (5.4%)	20 (37.7%)	<0.001
Reperfusion factors	–	–	–
Anterior circulation infarction (case)	165 (75.3%)	42 (79.2%)	0.528
Baseline NIHSS score	3 (2-4)	7 (4-12)	<0.001
DNT (min)	29 (24-36)	33 (26-45.5)	0.019
TOAST classification	–	–	<0.001
Large-artery atherosclerotic (case)	59 (26.9%)	28 (52.8%)	–
Small-artery occlusion (case)	122 (55.7%)	11 (20.7%)	–
Cardioembolism	24 (10.9%)	12 (22.6%)	–
Stroke of other determined etiology (case)	11 (5.0%)	1 (1.8%)	–
Stroke of undetermined etiology (case)	3 (1.3%)	1 (1.8%)	–

TIA, Transient Ischemic Attack; DNT, door to needle time; ONT, onset to needle time; DPT, door to puncture time; TOAST, Trial of Org 10172 in Acute Stroke Treatment; mRS, modified Rankin Scale score; NIHSS, National Institutes of Health Stroke Scale.

### 3.4 Multivariate analysis of factors influencing functional outcomes in patients undergoing Intravenous thrombolysis for AIS

After adjusting for confounding factors such as age, underlying diseases, comorbidities, and neurological function scores, it was found that delirium was significantly associated with poor functional prognosis 90 days after cerebral infarction, and was a strong independent risk factor (OR = 12.356, 95% CI 5.892 to 26.008, P < 0.001). At the same time, increasing age, hypertension, multiple comorbidities after admission (electrolyte imbalance, anemia, hypoproteinemia, etc.), increased baseline NIHSS score, and TOAST classification as large artery atherosclerosis type/cardiogenic embolism type would also significantly increase the risk of poor functional prognosis 90 days after cerebral infarction in patients (**Table 4**).

**Table 4:** Multivariate logistic regression analysis of independent predictors of poor 90-day functional outcomes in patients with AIS undergoing Intravenous thrombolysis.

Variable	OR	95% CI	*p*-value
Delirium	12.356	5.892-26.008	<0.001
Age	1.042	1.008-1.077	0.015
Sex	1.896	0.872-4.125	0.105
Illiterate	1.763	0.829-3.745	0.138
Hypertension	2.108	1.012-4.392	0.046
Coronary heart disease/atrial fibrillation	2.056	0.898-4.715	0.088
Electrolyte and metabolic disturbance	2.895	1.368-6.126	0.006
Anemia	3.102	1.458-6.605	0.003
Hepatic insufficiency	3.896	1.528-9.985	0.005
Renal insufficiency	2.785	1.096-7.078	0.031
Hypoproteinemia	3.205	1.536-6.690	0.002
Infection	3.598	1.689-7.665	0.001
Dysphagia	4.102	1.958-8.635	<0.001
The use of sedative drugs	3.892	1.798-8.435	0.001
Baseline NIHSS score	1.186	1.125-1.251	<0.001
DNT	1.012	0.998-1.026	0.085
TOAST classification (0 = small artery occlusion)	**-**	**-**	**-**
Large-artery atherosclerotic	2.985	1.468-6.072	0.003
Cardioembolism	2.689	1.256-5.752	0.011
Stroke of undetermined etiology	0.895	0.112-7.168	0.915
Stroke of other determined etiology	1.596	0.198-12.852	0.668

Variables with p < 0.1 in the univariate analysis were included in the logistic regression analysis. TOAST, Trial of Org 10172 in Acute Stroke Treatment; mRS, modified Rankin Scale score; NIHSS, National Institutes of Health Stroke Scale.

### 3.5 Multivariate analysis of poor outcomes 90 days after intravenous thrombolysis (model 1)

**Figure 2:**
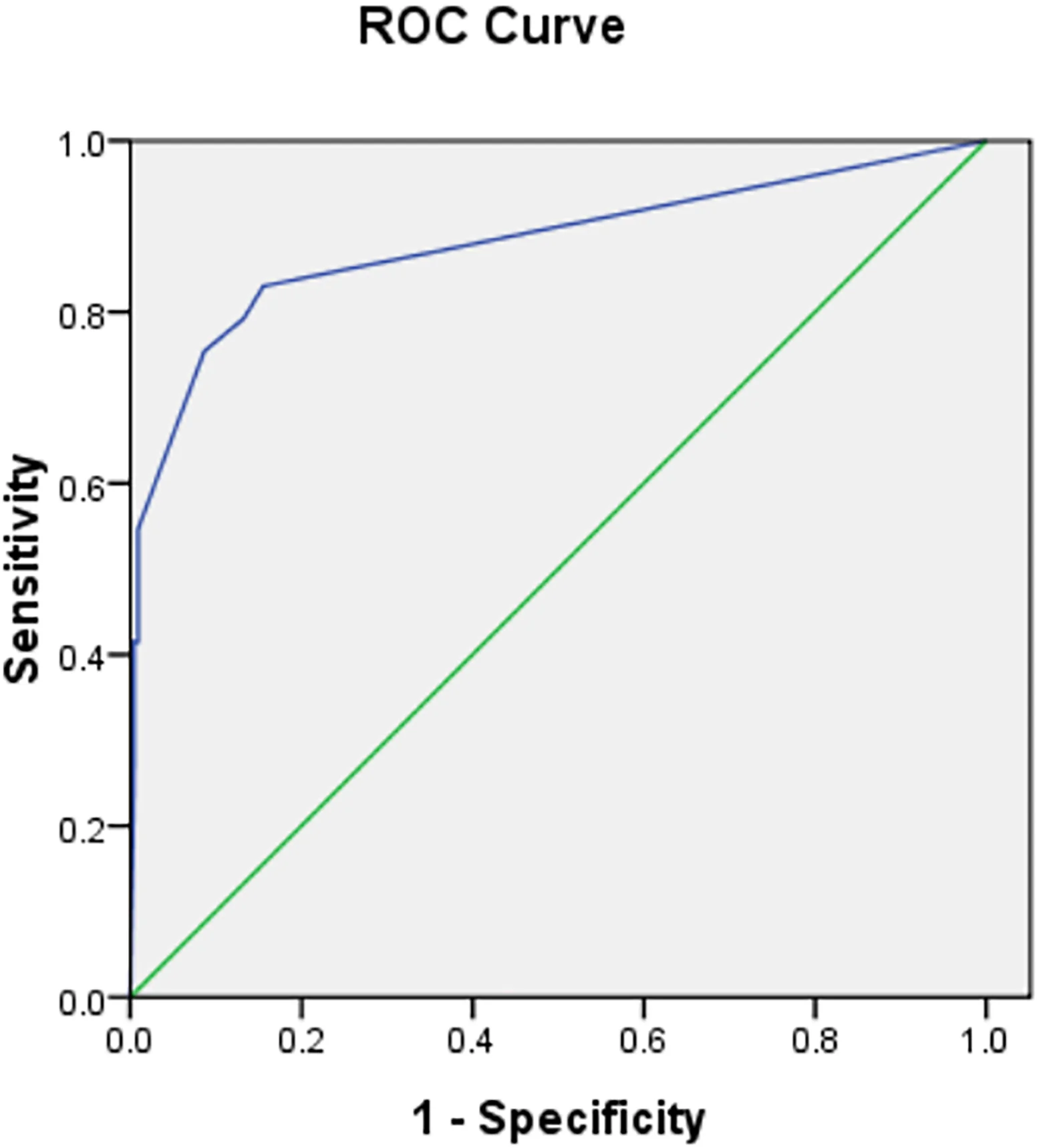
The ROC curve of the prediction for 90d poor outcomes.

Among the factors that significantly affected the 90-day prognosis (P < 0.01), those included delirium, age, baseline NIHSS score, electrolyte imbalance, anemia, liver dysfunction, hypoproteinemia, infection, dysphagia, and the use of sedative drugs were incorporated into Model 1 for ROC curve analysis to assess the risk of 90d poor outcomes. The ROC curve for predicting 90d poor outcomes after intravenous thrombolysis had an AUC of 0.880 [95% CI (0.816-0.944), P < 0.001] (**Figure 2**).

## 4 Discussion

Delirium represents a common complication following stroke, with the reported incidence of PSD varying substantially across studies (13, 17). Lawson et al. conducted a comprehensive literature review and reported that the incidence of PSD ranges from 12% and 75% (18). In the present analysis, 272 patients were enrolled, among whom 42 developed delirium during hospitalization, corresponding to an incidence rate of 15.4%, which aligns with previous findings.

The current findings showed that baseline NIHSS scores were independent risk factors for both the development of delirium and poor 90-day outcomes following intravenous thrombolysis in patients with AIS. Previous investigations have also reported that severe neurological deficits act as independent risk factors for PSD, which is consistent with these results (11, 19). As delirium constitutes an acute neuropsychiatric syndrome, severe neurological impairment often compromises an individual’s awareness of self and environment, thereby increasing susceptibility to delirium (20). Infection has been previously identified as an independent risk factor for delirium (21, 22). Consistent with these observations, the present analysis revealed that secondary infection during hospitalization was independently associated with the onset of delirium. It has been postulated that infection with pathogenic microorganisms induces the systemic release of inflammatory cytokines, which disrupt blood–brain barrier integrity. The translocation of inflammatory mediators into the central nervous system can activate microglia, promote the release of pro-inflammatory factors, and subsequently exert neurotoxic effects that contribute to delirium pathogenesis (23–25).

Dysphagia, a relatively frequent manifestation among individuals with AIS, has only recently been linked to delirium risk. A retrospective study by Cheng et al. of hospitalized patients in the intensive care unit demonstrated a significant association between dysphagia and delirium development (26). Consistent with these findings, the present analysis identified dysphagia as an independent risk factor for delirium. One potential explanation involves dysphagia-induced gastrointestinal dysfunction, which may disrupt the gut–brain axis, initiate inflammatory cascades, and alter neurotransmitter balance, thereby heightening vulnerability to delirium (27, 28). Furthermore, dysphagia may increase the risk of aspiration and subsequent pulmonary infection, triggering systemic inflammation that adversely affects the central nervous system and accelerates delirium onset (27).

Advancing age is widely recognized as an independent risk factor for PSD. Age-related physiological decline and the accumulation of comorbidities render older adults more vulnerable to external stressors, predisposing them to delirium (29). Additionally, neuronal apoptosis and the resultant reduction in cholinergic reserves with aging may contribute to the underlying pathophysiology (30). In this analysis, age was also identified as an independent risk factor for PSD, consistent with existing evidence. Previous literature has shown that parameters such as DNT influence delirium risk following reperfusion (31); However, our study did not yield such results. We speculate that this might be related to the following factors: (1) The sample size included was relatively small; (2) Although DNT is an important time indicator for intravenous thrombolysis, the occurrence of delirium may be influenced by many other factors, such as the location, size, collateral circulation status, and postoperative complications of cerebral infarction. These reperfusion time indicators are just one of the many influencing factors, and their effect is relatively small. Therefore, it was difficult to separately demonstrate the correlation with delirium in the study; (3) The patients included in the study generally had shorter DNTs, which may not reflect the impact of DNT duration on delirium. Despite the lack of association between DNT and delirium, prolonged DNT was identified as an independent predictor of poor 90-day functional outcomes. Longer DNT may lead to extended cerebral ischemia, increasing the likelihood of reperfusion injury and complications such as cerebral edema and hemorrhagic transformation. These pathophysiological processes may hinder neurological recovery and contribute to unfavorable outcomes, consistent with findings reported by Man et al. (32).

Analysis based on the TOAST classification revealed that, compared with small-artery occlusion, large-artery atherosclerosis was more frequently associated with both delirium and poor prognosis. This may be attributable to the underlying pathophysiology, as small-artery occlusion typically involves smaller penetrating vessels, resulting in limited infarct volume and milder symptoms. Conversely, large-artery atherosclerosis often affects cortical regions, producing larger infarct areas and more severe neurological deficits that predispose patients to altered consciousness.

Delirium has been closely associated with adverse prognoses in patients with ischemic stroke. Previous evidence from domestic and international studies has shown that patients with stroke who develop delirium have higher in-hospital mortality and elevated mortality rates within 12 months after stroke onset (5, 33, 34). Similarly, in this analysis, delirium is highly correlated with the risk of short-term death. Considering the possibility of delirium suggests that there may still be severe brain dysfunction after intravenous thrombolysis, which affects the recovery of neurological functions and increases the risk of disability. In our study, it was also found that delirium is a strong predictor of 90d poor outcomes in patients undergoing intravenous thrombolysis. From a mechanistic perspective, thrombolytic therapy not only rescues the ischemic penumbra but also damages the blood-brain barrier through inflammatory, oxidative stress, and neurotoxic pathways, triggering neuroinflammation and imbalance of neurotransmitters, thereby disrupting the thalamo-cortical network function and triggering delirium (4). Schäbitz et al. (2023) conducted a 2.5-year follow-up of 1141 patients with intravenous thrombolysis for AIS and found that post-stroke delirium was a significant negative predictor of good functional outcome (35). A large-sample study published in 2026 further confirmed that after multivariate correction, the risk of 3-month poor functional outcome (mRS > 2) in patients with post-stroke delirium increased by 3.36 times (OR = 3.362, 95% CI 1.851–6.107), and this association was independent of complications such as post-stroke pneumonia and urinary tract infections (36). All these studies are consistent with the results of this study.

Understanding the relationship between delirium and outcomes of intravenous thrombolysis is helpful for clinicians to strengthen the early identification of delirium in ischemic stroke patients after intravenous thrombolysis. For patients who develop delirium after intravenous thrombolysis, doctors can adjust the treatment plan according to the severity and cause of delirium, and can more accurately predict the possible adverse prognosis of the patients, thereby conducting more adequate communication with the patients’ families and formulating more reasonable rehabilitation plans and care plans.

Several limitations should be acknowledged. First, the study was conducted at a single stroke center with relatively small subgroup sample sizes, which may limit statistical power and introduce bias. Future multicenter studies with larger samples are warranted to improve generalizability. Second, the study design and institutional constraints limited the inclusion of certain potentially relevant clinical indicators, which may have influenced the findings. Third, due to limitations in the delirium assessment instruments, patients with severe aphasia or persistent coma were excluded, which may have affected the comprehensiveness of the results. Fourth, there is a complex interaction between delirium and the severity of brain injury, and a purely cross-sectional analysis is unable to fully establish a causal relationship. Fifth, the proportion of male patients in the research cohort is excessively high (62%), which may limit the general applicability of our research results to female stroke patients. Sixth, this study did not systematically collect the pre-stroke functional status (such as pre-stroke mRS), which is a strong predictor of stroke prognosis. Although our cohort (patients receiving intravenous thrombolysis treatment) may have potential screening for the pre-stroke functional status and relevant factors such as age and comorbidities have been corrected, there may still be residual confounding. Future studies should include detailed pre-stroke functional assessment to more accurately estimate the independent effect of delirium.

Delirium is a strong risk factor for poor 90-day prognosis, and its early identification is of great clinical significance. Patients who develop delirium should all be regarded as high-risk individuals for poor prognosis and require more intensive monitoring and more active rehabilitation interventions. Effective management of delirium can shorten hospital stay and reduce mortality. Therefore, including delirium in the prognostic assessment system means that clinicians can shift from passive prediction to active intervention.

Specific intervention measures for delirium include non-pharmacological interventions (such as cognitive orientation, minimization of restraint, pain management, avoidance of the use of sedative drugs, early activities and family participation, etc.), sleep management (reducing unnecessary nursing operations at night, reducing ward noise and light, using eye masks and earplugs, and avoiding the use of sedative-hypnotic drugs as much as possible), and pharmacological interventions (for patients with severe restlessness that may endanger life safety or affect treatment, small doses of antipsychotic drugs such as haloperidol can be considered, and benzodiazepines should be avoided as much as possible) (37, 38).

This study is a retrospective study. Due to the limitations of medical record data and inclusion criteria, key variables such as pre-stroke mRS, vascular reperfusion status, hemorrhage transformation, infarction volume, mechanical thrombectomy, and occlusion sites were not included in the analysis. There are unavoidable residual confounding, which may affect the stability of the association results between delirium and 90-day functional outcome. Further verification is needed in prospective studies.

## 5 Conclusions

Delirium is significantly associated with poor functional outcomes at 90 days in patients with AIS treated with intravenous thrombolysis, highlighting the clinical importance of early delirium detection. Dysphagia, secondary infections, hypoproteinemia, high baseline NIHSS scores, and large-artery atherosclerotic and cardioembolic stroke subtypes are independently associated with both delirium occurrence and adverse 90-day outcomes.

## Data Availability

The raw data supporting the conclusions of this article will be made available by the authors, without undue reservation.
